# Advances in Expression Regulation, Molecular Targeting Mechanisms, and Therapeutic Applications of the Let-7 MicroRNA Family in Gastric Cancer

**DOI:** 10.32604/or.2025.067546

**Published:** 2025-11-27

**Authors:** Xinke Chai, Shifeng Wu, Qian Shen, Qiulin Huang

**Affiliations:** The First Affiliated Hospital, Department of Gastrointestinal Surgery, Hengyang Medical School, University of South China, Hengyang, 421001, China

**Keywords:** Stomach neoplasms, microRNAs, Let-7, carcinogenesis, biomarkers

## Abstract

Gastric cancer (GC) is a prevalent malignant tumor globally, with high incidence and mortality rates. Advances in understanding molecular mechanisms underlying GC have highlighted the role of microRNAs (miRNAs) in its initiation, progression, and treatment. The Let-7 family, an important class of miRNAs, is closely associated with the biological behaviors of GC. Aberrant expression of various Let-7 family members in GC patients contributes to disease progression, as they target multiple molecular pathways and participate in diverse regulatory mechanisms throughout GC pathogenesis. This article systematically summarizes the expression patterns of Let-7 family members in GC, explores their influence on GC cell behaviors such as proliferation, invasion, and metastasis through key target gene regulation, and reviews current advances in Let-7-based interventions for GC treatment. It aims to provide foundational insights for a deeper understanding of Let-7-related mechanisms in GC and optimize therapeutic strategies.

## 1 Introduction

Gastric cancer (GC) is the fifth most prevalent cancer globally and ranks as the fifth leading cause of cancer-related deaths [1]. The majority of patients are diagnosed at advanced stages, which significantly restricts the available treatment options and leads to a poor prognosis [2,3]. GC is characterized by high recurrence, metastasis, and mortality rates, especially in cases with distant spread, where the five-year survival rate is alarmingly low, posing a serious risk to health and life [4]. Conventional treatment strategies for GC mainly consist of surgical removal, chemotherapy, and radiation therapy [4,5]. Although surgical intervention is the primary method for treating GC, its effectiveness diminishes in advanced cases [6,7]. Chemoradiotherapy can serve as an adjuvant to surgery, either enhancing the therapeutic effect before the operation or complementing post-surgical treatment to address potential residual lesions; however, its efficacy is often limited and highly variable, frequently accompanied by a range of adverse events [8,9]. As research into the mechanisms underlying GC progresses, biomolecular therapy has gained attention as a significant area of focus. Although this novel cancer treatment method is gradually becoming a crucial component of GC management, its development continues to encounter numerous challenges [7,10]. Key challenges in the realm of biomolecular therapy for GC include the absence of specific tumor markers, the relative delay in the advancement of molecular targeted therapy compared to other malignancies (such as lung and breast cancer), and the issue of patients developing resistance during biomolecular therapy [11–13]. Overcoming these obstacles is imperative for improving treatment outcomes for GC.

Recent investigations into the molecular processes involved in GC have increasingly highlighted the role of microRNAs (miRNAs), which are small non-coding RNAs that play a crucial regulatory role in tumorigenesis, development, angiogenesis, and metastasis [14,15]. These miRNAs exert their influence by binding to complementary sites in the 3^′^-untranslated region (3^′^-UTR) of specific mRNAs, leading to the inhibition of gene translation or promoting mRNA degradation, thus affecting the behavior of cancer cells [15,16]. Notably, existing studies have demonstrated that the expression of Let-7, an important member of the miRNA family, is aberrant in GC, and this abnormal expression is closely associated with disease progression and patient prognosis [17,18]. A thorough investigation into the Let-7 family’s role and mechanisms in GC not only deepens our comprehension of the disease’s origins but also has the potential to identify new biomarkers and therapeutic targets for early detection, targeted treatment, and prognosis, ultimately contributing to the improvement of patient survival outcomes and the enhancement of their quality of life.

## 2 Overview of the Let-7 Family

### 2.1 Classification and Biosynthesis of the Let-7 Family

**Figure 1:**
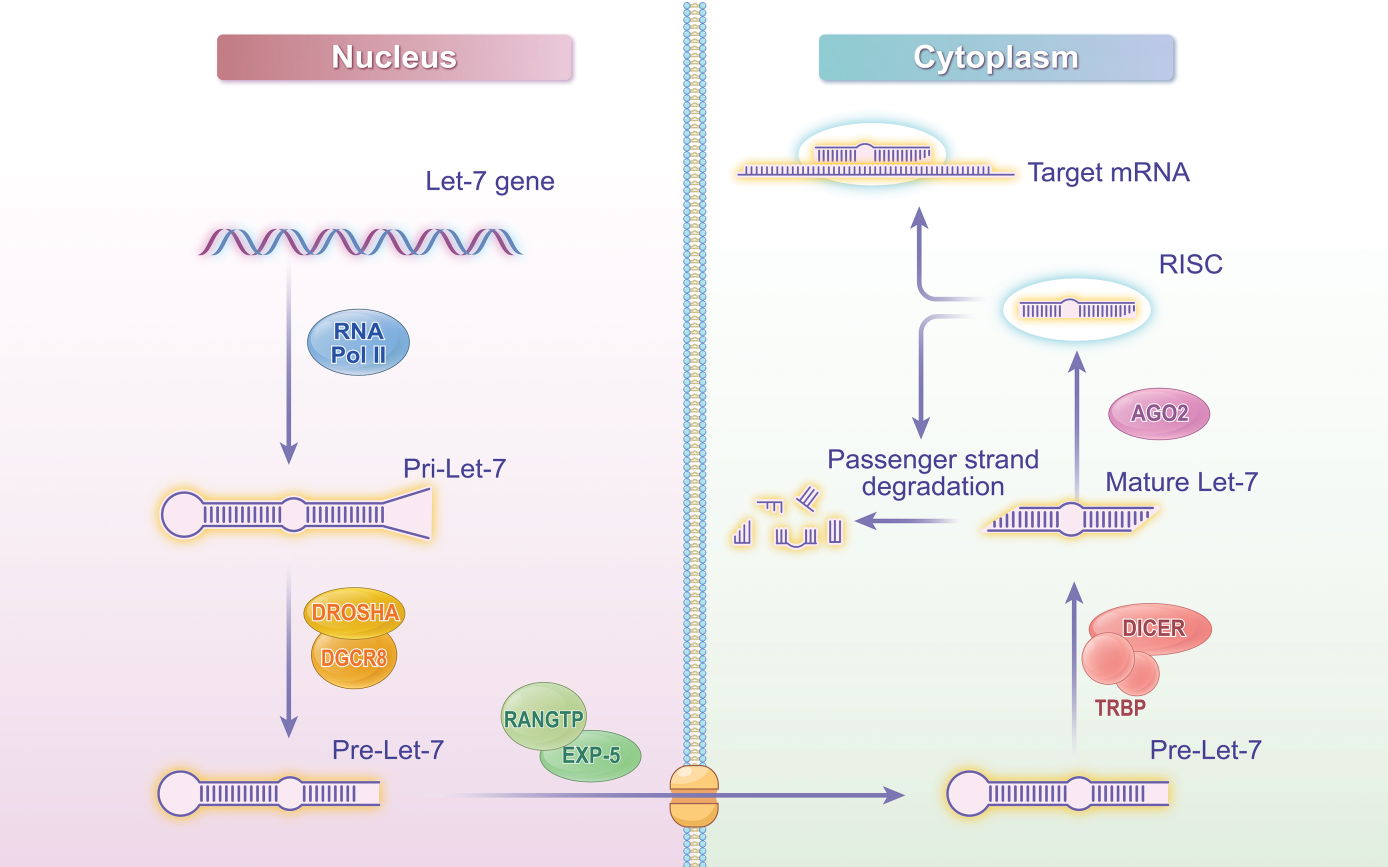
Let-7 biogenesis schematic diagram. Created with adobe illustrator

Most miRNAs feature a conserved sequence of 2–8 nucleotides, referred to as the seed “sequence”, which facilitates their categorization into various families. Among these, members with the seed sequence “T G A G G T A” are categorized into the Let-7 family [19,20]. Let-7, recognized as the first miRNA identified in humans, exhibits a high degree of conservation across different species [21,22]. The biosynthesis of Let-7 has been extensively studied within miRNA research. Initially, RNA polymerase II (Pol II) transcribes the primary Let-7 (Pri-Let-7). Subsequently, Pri-Let-7 is cleaved in the nucleus by the microprocessor complex, which comprises the Drosha enzyme and its cofactor DiGeorge Syndrome Critical Region 8 (DGCR8), resulting in the generation of precursor Let-7 (Pre-Let-7). Transport proteins, including Exportin-5 and Ran guanosine triphosphatase (Ran-GTPase), translocate Pre-Let-7 to the cytoplasm through the nuclear pore complex. In the cytoplasm, the enzyme Dicer and its cofactor TAR RNA binding protein (TRBP) further cleave and process Pre-Let-7 to form a double-stranded Let-7 molecule, consisting of a guide strand and a passenger strand. The guide strand is loaded onto the Argonaute protein to form the RNA-induced silencing complex (RISC). This complex can recognize target sequences and degrade the passenger strand. The mature Let-7-RISC complex binds to the 3^′^-UTR region of the target mRNA, leading to the degradation of the target mRNA or inhibition of its translation [23–26]. Through this mechanism, Let-7 effectively regulates the expression level of the target mRNA, thereby playing a crucial regulatory role within the cell and influencing cellular biological functions and signaling pathways [23,24,27,28]. The detailed process is illustrated in Fig. 1.

This figure illustrates the biosynthesis process of Let-7 microRNA, depicting key steps from transcription to the formation of the mature functional complex. The process begins with the transcription of the let-7 gene by Pol II in the nucleus, generating pri-let-7. The microprocessor complex, consisting of Drosha and DGCR8, cleaves pri-let-7 to produce pre-let-7. Pre-let-7 is then transported to the cytoplasm via EXP-5 in a Ran-GTP-dependent manner. In the cytoplasm, Dicer and TRBP further process pre-let-7 into a double-stranded molecule, from which the guide strand is loaded onto AGO2 to form the RISC. The mature let-7-RISC complex binds to the 3^′^-UTR of target mRNAs, leading to mRNA degradation or translational inhibition. Abbreviations: Pol II (RNA polymerase II), DGCR8 (DiGeorge Syndrome Critical Region 8), EXP-5 (Exportin-5), Ran-GTPase (Ras-related nuclear protein GTPase), TRBP (TAR RNA-binding protein), RISC (RNA-induced silencing complex), Ago (Argonaute), 3^′^-UTR (3^′^-untranslated region).

### 2.2 Members and Functions of the Let-7 Family

MiRNAs within the same gene cluster frequently exhibit high sequence homology. To differentiate between the highly similar members of the Let-7 family, letter suffixes are typically appended to their names (e.g., Let-7a, Let-7b). The distinctions represented by these letter suffixes often correspond to subtle variations in nucleotide sequences, suggesting that while they are generally homologous, they may possess differing functional characteristics. In contrast, when pre-miRNAs originating from different chromosomal loci produce the same mature miRNA sequence, they are differentiated by appending Arabic numeral suffixes to their designations. For instance, Let-7a-1 and Let-7a-2 are precursors located on distinct chromosomes, yet both generate the mature sequence Let-7a [29].

In humans, there are a total of 10 mature Let-7 family sequences (Let-7a, Let-7b, Let-7c, Let-7d, Let-7e, Let-7f, Let-7g, Let-7i, miR-98, and miR-202), derived from 13 Pre-Let-7 expressions [29,30]. Pre-Let-7, located at different chromosomal sites, can yield the same mature Let-7 sequence. As an example, three separate pre-Let-7 genes give rise to the identical mature Let-7a sequence. To identify these specific precursor transcripts, they are designated as Let-7a-1, Let-7a-2, and Let-7a-3. Similarly, the mature Let-7f sequence is produced by two distinct pre-Let-7 genes, which are consequently named Let-7f-1 and Let-7f-2 [29–31].

Let-7 plays a pivotal role in various essential physiological and pathological processes within organisms. In embryonic development, Let-7 regulates cell lineage differentiation [32,33]. In metabolic homeostasis, it finely modulates the metabolism of sugars, lipids, and amino acids [34–36]. Within the cardiovascular system, Let-7 is involved in the proliferation and differentiation of cardiomyocytes, as well as the regulation of angiogenesis [37,38]. In the field of neurology, it protects neurons and delays the progression of neurodegenerative diseases [39–42]. Additionally, in immune regulation, Let-7 bidirectionally controls the differentiation and function of immune cells [43–45]. Furthermore, Let-7 holds significant importance in oncology, often classified as a tumor suppressor that reduces tumor invasiveness and is closely associated with tumor resistance to radiotherapy and chemotherapy. It influences cancer development by targeting oncogenes and stem cell factors, as well as regulating related target genes involved in the cell cycle and proliferation, thereby demonstrating its substantial value in cancer prevention and treatment [46–48].

## 3 Expression of the Let-7 Family in GC

The expression profiles of miRNAs in tumor tissues exhibit significant variability, with miRNA signatures closely linked to specific clinical and biological characteristics [49,50]. While a general downregulation of Let-7 family members has been observed across various malignant tumors, research indicates that, under certain conditions, the expression of specific Let-7 family members can be upregulated in these tumors [51,52]. This variability in expression patterns is also evident in GC, where different Let-7 family members display distinct expression levels, and even the same Let-7 family member may demonstrate varying expression trends under different conditions. To achieve a comprehensive understanding of the expression patterns and variations of the Let-7 family in GC, we systematically summarized the expression profiles of the Let-7 family in GC (Table 1).

**Table 1:** The expression status of Let-7 family members in GC

Family members	Expression changes	Source of detection	Analysis platform	References
Let-7a	Downregulation	Plasma	qRT-PCR	Tsujiura et al., 2010 [53]
	Downregulation	Gastric cancer tissue	ISH	Zhu et al., 2010 [54]
	Downregulation	Gastric cancer tissue	qRT-PCR	Yang et al., 2011 [55]
	Downregulation	Serum	qRT-PCR	Song et al., 2012 [56]
	Upregulation^a^	Gastric cancer tissue	BIAMDV	Liu et al., 2021 [57]
	Downregulation	Gastric cancer tissue	qRT-PCR	Sallas et al., 2021 [58]
	Downregulation	Gastric cancer tissue	qRT-PCR	Cholewinski et al., 2022 [59]
Let-7b	Downregulation	Gastric cancer tissue	qRT-PCR	Sallas et al., 2021 [58]
	Downregulation	Gastric cancer tissue	qRT-PCR	Kang et al., 2014 [60]
	Downregulation	Gastric cancer tissue	qRT-PCR	Han et al., 2015 [61]
	Downregulation	Gastric cancer tissue	qRT-PCR	Yu et al., 2015 [62]
Let-7c	Downregulation	Gastric cancer tissue	qRT-PCR	Fassan et al., 2016 [63]
Let-7d	Upregulation^b^	Gastric cancer tissue	qRT-PCR	Gao et al., 2022 [64]
Let-7f	Downregulation	Gastric cancer tissue	qRT-PCR	Liang et al., 2011 [65]
	Upregulation	Serum	qRT-PCR	Liu et al., 2015 [66]
	Upregulation	Plasma	qRT-PCR	Peng et al., 2018 [67]
Let-7g	Downregulation	Gastric cancer tissue	qRT-PCR	Kang et al., 2014 [60]
	Downregulation^c^	Gastric cancer tissue	qRT-PCR	Tang et al., 2020 [68]
	Upregulation^c^	Serum exosomes	qRT-PCR	Tang et al., 2020 [68]
Let-7i	Upregulation	Serum	qRT-PCR	Liu et al., 2015 [66]
	Downregulation	Gastric cancer tissue	qRT-PCR	Liu et al., 2012 [69]
	Upregulation	Gastric cancer tissue	qRT-PCR	Treece et al., 2016 [70]
	Upregulation	Gastric cancer tissue	BIAMDV	Yuan et al., 2019 [71]
	Upregulation^d^	Gastric cancer tissue	qRT-PCR	Codolo et al., 2019 [72]
	Downregulation	Gastric cancer tissue	qRT-PCR	Shi et al., 2019 [73]
miR-98	Upregulation	Gastric cancer tissue	qRT-PCR	Yao et al., 2009 [74]
	Downregulation^e^	Gastric cancer tissue	qRT-PCR	Xu et al., 2021 [75]
	Downregulatione	Gastric cancer tissue	qRT-PCR	Zhang et al., 2024 [76]
miR-202	Downregulation^f^	Gastric cancer tissue	qRT-PCR	Zhao et al., 2013 [77]
	Upregulation^f^	Type 1 gastric neuroendocrine tumor tissue	qRT-PCR	Dou et al., 2018 [78]

Note: ^a^Let-7a-3p; ^b^Let-7d-5p; ^c^Let-7g-5p; ^d^Let-7i-5p; ^e^mir-98-5p; ^f^miR-202-3p; qRT-PCR: Quantitative reverse transcription polymerase chain reaction; ISH: *In situ* hybridization; BIAMDV: Bioinformatics integrated analysis and multi-database validation.

### 3.1 Members of the Let-7 Family Showed Generally Low Expression in GC

Most studies have found that members of the Let-7 family generally exhibit a downregulated expression trend in tumor tissues, plasma, and serum of GC patients. Among them, Let-7a, a typical tumor suppressor, shows significant downregulation in GC patients, with experimental data confirming that its overexpression can induce apoptosis in GC cells [55,79]. Furthermore, the expression level of Let-7a gradually decreases as the gastric mucosa progresses from normal to cancerous, reflecting the tumor differentiation stage in GC patients [55]. Studies have demonstrated a significant association between Let-7a and GC metastasis: its expression level is notably lower in GC tissues with lymph node metastasis compared to those without [54]. Similarly, other members of the Let-7 family, such as Let-7b, Let-7c, Let-7g, and miR-202, are generally downregulated. These molecules, which share similar functions with Let-7a, are closely related to poor prognosis and lymph node metastasis, and they inhibit the growth, invasion, and migration of GC cells while inducing their apoptosis [60–62,76,77].

### 3.2 Expression Differences of Pre-Let-7 Arm End Splicing Subtypes

Pre-Let-7 can generate mature Let-7 from either its 5^′^ or 3^′^ arm. Notably, Let-7 derived from different arms may exhibit distinct expression patterns. Specifically, while Let-7a generally functions as a tumor suppressor and shows an overall downregulation trend in GC, studies have indicated that Let-7a-3p (produced from the 3^′^ arm cleavage of Pre-Let-7a) is actually upregulated in GC tissues [57]. The expression of miR-98 and its isoforms in GC also demonstrates variability. Although miR-98 is typically upregulated in GC tissues, miR-98-5p (derived from the 5^′^ arm of Pre-miR-98) exhibits a downregulation trend, with studies showing that miR-98-5p can inhibit the proliferation, migration, and invasion capabilities of GC cells [74–76]. Additionally, the expression of Let-7i and its isoform Let-7i-5p in samples from GC patients also reflects diversity. Multiple studies have reported the upregulation of Let-7i and Let-7i-5p expression in GC tissues or serum [66,70–72]. Significantly, some studies have also noted the downregulation of Let-7i, suggesting that increased expression of Let-7i may inhibit the proliferation, metastasis, and invasion of cancer cells. Conversely, decreased expression levels of Let-7i are closely associated with shorter survival, local invasiveness, and lymph node metastasis [69,73].

### 3.3 Expression Differences of the Let-7 Family in Distinct Tissue Sources of GC

Let-7 exhibits differential expression in different sample types from GC patients. Specifically, Let-7f expression detected in serum and plasma samples of GC patients generally shows an upregulation trend [66,67], whereas its expression in GC tissue samples demonstrates a downregulation trend and is associated with inhibition of cell invasion and metastasis [65]. This expression discrepancy is also observed in Let-7g-5p: its expression is upregulated in serum exosomes of early-stage GC patients but significantly downregulated in tissue samples from early-stage GC patients [68]. However, some members of the Let-7 family maintain relatively consistent expression patterns across different tissues in GC patients. For example, a study comparing Let-7a expression in malignant tissues of the gastric body versus the gastric antrum found no statistically significant difference in expression levels between the two [59].

### 3.4 Expression Differences across Tumor Types and Histological Subtypes of GC

The expression profile of Let-7 exhibits significant variations among different tumor tissue types and across various stages of the same tumor type. For instance, miR-202-3p is downregulated in GC tissues and has been demonstrated to inhibit cell proliferation and induce apoptosis in both *in vitro* and *in vivo* experiments [77]. In contrast, in type 1 gastric neuroendocrine tumor tissues, the expression of miR-202-3p is upregulated [78]. Studies of GC histological subtypes reveal that miR-202 is upregulated in intestinal-type GC compared to diffuse-type GC [80]. Furthermore, differences in Let-7 expression have been observed between signet ring cell carcinoma and tubular adenocarcinoma, with research indicating that Let-7i expression is higher in signet ring cell carcinoma than in tubular adenocarcinoma [81].

### 3.5 The Association between Let-7 Family Expression and Molecular Subtypes of GC

The Cancer Genome Atlas (TCGA) classified GC into four molecular subtypes: Epstein-Barr virus-positive (EBV), microsatellite instability-high (MSI-H), chromosomal instability (CIN), and genomically stable (GS) [82,83]. Research reveals distinct Let-7 miRNA expression patterns across these subtypes. Some analyses demonstrate reduced Let-7c-5p expression in MSI-H subtype [84]. For EBV-positive tumors, some Let-7 family members (Let-7a and Let-7f) show marked expression downregulation linked to tumor progression [85].

Despite these advances, systematic investigations correlating GC molecular subtypes with Let-7 expression remain limited. Future in-depth and comprehensive studies are necessary to clarify these relationships, to thoroughly explore the expression profiles of the Let-7 family across all molecular subtypes, and their pathological significance.

### 3.6 Causes of Expression Differences

This article presents several reasons for the observed discrepancies. First, functional differences exist among members of the Let-7 family and across different subtypes, suggesting that Let-7 regulatory mechanisms may be specific to individual members and subtypes. Second, significant variations in miRNA absorption and degradation efficiency across various tissues contribute to differences in Let-7 expression characteristics. Third, the distinct microenvironments of different GC subtypes may influence miRNA biological functions. Fourth, variations in technology and sequencing platforms may also contribute to the differential expression of Let-7 family members in GC.

Given that the dysregulation of Let-7 expression has been linked to the onset and progression of GC, it is reasonable to consider Let-7 family members as potential prognostic, diagnostic, and therapeutic indicators in GC treatment. A more profound understanding of Let-7’s differential expression during GC development and progression may provide new insights for its clinical application.

### 3.7 Clinical Significance of Expression Differences and Bioinformatics Models

The differential expression of miRNAs serves as a critical indicator for the clinical diagnosis and prognostic assessment of GC [86,87]. The incorporation of bioinformatics and machine learning models has significantly improved the precision of risk stratification and the depth of mechanistic interpretation [88,89]. Numerous studies integrating bioinformatics predictions with experimental validation have systematically demonstrated the potential value of Let-7 family members in the progression and therapy of GC, a finding corroborated in large-scale clinical cohorts, underscoring their translational application potential.

Bioinformatics and machine learning models have emerged as powerful tools for screening biomarkers of the Let-7 family. For instance, Let-7a-3p [57], the circulating miR-106a/Let-7a ratio [53], and serum exosomal Let-7g-5p [68]—identified through algorithmic screening and clinically validated—demonstrate superior diagnostic efficacy. Their area under the curve (AUC) values are significantly higher than those of traditional tumor biomarkers, providing novel strategies for the non-invasive diagnosis of GC. Furthermore, members of the Let-7 family exhibit prognostic relevance. An integrative bioinformatics analysis, combined with functional experiments, revealed that low expression of Let-7i serves as an independent poor prognostic factor, closely associated with the aberrant activation of genes that drive tumor invasion and metastasis [69].

Clinical translational studies have confirmed the application value of several markers from the Let-7 family, including their use in early diagnostic panels, prognostic prediction models, and therapeutic response assessment indicators. However, current research still encounters challenges, such as addressing expression heterogeneity, optimizing population-specific models, and standardizing detection methods. Future studies should prioritize multi-omics integrated analysis and single-cell level mechanistic interpretation to offer more comprehensive theoretical support for the precision diagnosis and treatment of GC.

## 4 Related Targets and Mechanisms of Action of the Let-7 Family in GC

By analyzing the evolutionary sequence conservation between nematodes and humans, researchers have discovered that Let-7 specifically targets RAS in human cancer cells [90–92]. This finding has led to increasing recognition that members of the Let-7 family influence the occurrence and progression of cancer by regulating a series of key targets. In recent years, research on the role of Let-7 in GC has progressed significantly. To gain a more comprehensive understanding of the specific associations between Let-7 and its targets in the occurrence and development of GC, we have summarized the target genes and their roles, along with the corresponding regulatory mechanisms, of Let-7 family members in GC (Table 2).

**Table 2:** The relevant target sites of Let-7 family members in GC

Target pathway	Research subjects	References
↓Let-7a → ↑RAB40C → ↑Proliferation and anchorage-independent growth of GC cells.	Human gastric epithelial cell line GES-1, human GC cell lines (AGS, SGC-7901, BGC-823, MKN-28, and HGC-27), and fresh GC tissue specimens.	Yang et al., 2011 [55]
↓Let-7a → ↑HMGA2 → ↑Serosal invasion and lymphovascular invasion ↓affect overall survival.	Human GC cell lines (MKN1, MKN28, MKN45, MKN74, KATOIII, and AZ521), fresh GC tissue specimens.	Motoyama et al., 2008 [93]
↓Let-7a → ↑C-Myc→↑hnRNPA1→↑PKM2→↑ Proliferation, migration, and invasion of GC cells.	Human gastric epithelial cell line GES-1, human GC cell lines (SGC7901, BGC823, MGC803), and fresh GC tissue specimens.	Tang et al., 2016 [94]
↓Let-7a → ↑Rictor → ↑AKT → ↑mTOR → ↓Autophagy in GC cells.	Human gastric epithelial cell line GES-1, human GC cell lines (MGC-803, SGC-7901, BGC-823, AGS).	Fan et al., 2018 [95]
↓Let-7a → ↑COPB2 → ↑Growth and invasion of GC cells, ↓GC cell apoptosis.	Human gastric epithelial cell line GES-1, human GC cell lines (SGC7901, MKN45, BGC823).	Chen et al., 2021 [96]
↓Let-7b/g → ↑AKT2 → ↑mTOR → ↑Growth, proliferation, and invasion of GC cells.	Human GC cell lines (MKN1, MKN7, MKN28, MKN45, SNU1, SNU16, AGS, KatoIII, NCI-N87).	Kang et al., 2014 [60]
↓Let-7b → ↑ING1 → ↑Invasion and metastasis of GC cells.	Human GC cell lines (GC9811, GC9811-p, SGC7901-NM, SGC7901-M) and fresh GC tissue specimens.	Han et al., 2015 [61]
↓Let-7b → ↑Cthrc1 → ↑Proliferation, migration, and invasion of GC cells.	Human GC cell lines SGC7901 and fresh GC tissue specimens.	Yu et al., 2015 [62]
↓Let-7b → ↑C-Myc → ↓Chemosensitivity of GC cells to chemotherapeutic drugs.	Human GC cells lines SGC7901, drug-resistant cells (SGC7901/DDP, SGC7901/VCR).	Yang et al., 2015 [97]
↓Let-7c → ↑ET-1 → ↑Proliferation, growth, and migration of GC cells.	Human GC cell lines (AGS, AZ-521, NUGC, TSGH).	Tsai et al., 2015 [98]
↓Let-7c-3p → ↑SOX2 → ↓Chemosensitivity of GC cells to chemotherapeutic drugs.	Human GC cell lines (HGC-27, NCI-N87).	Zhao et al., 2023 [99]
↑Let-7d-5p → ↓PRDM5 → ↑Proliferation, migration, and invasion of GC cells, ↓GC cell apoptosis.	Human GC cell lines (SGC-7901, AGS).	Gao et al., 2022 [64]
↓Let-7f → ↑MYH9 → ↑myosin IIA → ↑invasion and metastasis of GC cells.	Human GC cell lines (GC9811, GC9811-p, SGC7901-NM, SGC7901-M) and fresh GC tissue specimens.	Liang et al. 2011 [65]
↑Let-7g-3p → ↓RAPGEF3 → ↑TGF-β receptor TβRI → ↑metastasis and chemoresistance of GC cells.	Human GC cell lines (GES-1, AGS, NCI-N87) and fresh GC tissue specimens.	Luo et al., 2023 [100]
↓Let-7g-5p → ↓SOCS7 → ↑STAT3 phosphorylation → M2 polarization → ↑proliferation, growth, migration, invasion, and immune evasion of GC cells.	Human GC cell lines (MKN45, AGS) and fresh GC tissue specimens.	Ye et al., 2025 [101]
↓Let-7i → ↑COL1A1 → ↑Proliferation, migration, and invasion of GC cells.	Human GC cell lines (SGC-7901, MGC-803, AGS, N87) and fresh GC tissue specimens.	Shi et al., 2019 [73]
↓miR-98-5p → ↑IL-6 → ↑Treg/Th17 → ↑Immunotolerance of GC cells.	Fresh GC tissue specimens and peripheral blood specimens from GC patients.	Xu et al., 2021 [75]
↓miR-98-5p → ↑USP44 → ↑CTCFL → ↑Proliferation, migration, and invasion of GC cells.	Fresh GC tissue specimens.	Zhang et al., 2024 [76]
↓miR-202-3p → ↑Gli1 → ↑Proliferation of GC cells, ↓GC cell apoptosis.	Human GC cell lines (MKN-45, SGC-7901, AGS, NCI-N87, SNU-1, KATO III, BGC-823, MKN-28).	Zhao et al., 2013 [77]
↑miR-202-3p → ↓DUSP1 → ↑Growth of enterochromaffin-like cells.	Type 1 gastric neuroendocrine tumor tissue.	Dou et al., 2018 [78]

### 4.1 Related Targets Involved in Let-7 Regulation of Growth, Proliferation, Migration, and Invasion of GC

The Let-7 family members exhibit bidirectional regulatory characteristics in the development and progression of GC. Most members exert tumor-suppressive effects through multiple targets, inhibiting the growth, proliferation, migration, and invasion of GC cells. For instance, Let-7a binds to the 3^′^-UTR of RAB40C mRNA, negatively regulating this gene and downregulating hnRNPA1 and PKM2 by targeting C-Myc, thereby blocking glycolysis-dependent proliferation and invasion [55,94]. Concurrently, Let-7a downregulates HMGA2, further inhibiting the invasion of GC cells [93]. Let-7b/g targets the AKT/mTOR pathway to obstruct pro-cancer signaling, and their expression is negatively correlated in humans [60]. Additionally, Let-7b/c/f/i and miR-98-5p inhibit the proliferation, migration, invasion, and metastasis of GC cells by negatively regulating genes such as ING1/CTHRC1 [61,62], ET-1 [98], MYH9 [65], COL1A1 [73], and USP44 [76], respectively. In normal tissues, the Let-7 family maintains cellular homeostasis by negatively regulating oncogenes, while its downregulation in GC patients often leads to the dysregulation of oncogenic pathways.

However, certain members can promote the growth, proliferation, migration, and invasion of GC by inhibiting tumor suppressor genes. For instance, Let-7d-5p targets the tumor suppressor gene PRDM5 [64], Let-7g-3p targets RAPGEF3 [100], and miR-202-3p targets DUSP1 [78]. Collectively, these interactions accelerate the malignant transformation of GC cells by attenuating tumor suppressor signaling.

This bidirectional regulatory characteristic underscores the complexity of the Let-7 family within the molecular network of GC. Its function is dependent on specific family members, target genes, and microenvironmental variations, establishing it as both a tumor suppressor and a therapeutic target. A comprehensive analysis of its precise regulatory mechanisms will provide a more robust theoretical foundation for the precision stratification and targeted intervention of GC.

### 4.2 Related Targets Involved in the Regulation of Apoptosis and Autophagy in GC

Members of the Let-7 family play a significant role in influencing GC cell apoptosis and autophagy through the regulation of downstream targets. Let-7a can activate autophagy by targeting Rictor, which is an upstream molecule in the AKT-mTOR pathway [95]. Additionally, it inhibits proliferation and induces apoptosis by silencing the oncogene Coatomer protein complex subunit beta 2 (COPB2), thereby suppressing the receptor tyrosine kinase (RTK) signaling pathway [96]. Let-7d-5p targets the tumor suppressor gene PRDM5, which not only promotes GC cell proliferation, migration, and invasion but also reduces patient survival by inhibiting autophagy [64]. Furthermore, miR-202-3p directly targets Gli1, a key transcription factor in the Sonic Hedgehog (SHH) pathway, leading to downregulation of its target genes, γ-catenin and Bcl-2. This action inhibits GC cell growth and induces apoptosis both *in vitro* and *in vivo* [77].

These regulatory patterns illustrate the dual role of Let-7 family members in determining the fate of GC cells. They can exert tumor-suppressive effects by activating autophagy or inducing apoptosis, while also promoting tumor progression by inhibiting autophagy or interfering with apoptosis pathways. The complex molecular mechanisms provide a multi-dimensional theoretical foundation for elucidating the development of GC and for developing targeted therapeutic strategies.

### 4.3 Related Targets Involved in the Regulation of the Immune Microenvironment in GC

Some studies have revealed that members of the Let-7 family play a dual regulatory role in the immune microenvironment of GC. Specifically, miR-98-5p promotes anti-tumor immune activation by reshaping the balance between regulatory T cell (Treg) and Th17 cells [75], while Let-7g-5p facilitates immune escape through the induction of M2 macrophage polarization and the upregulation of immune checkpoint molecules [101]. This bidirectional regulatory mechanism underscores the complexity of the Let-7 family in tumor-immune interactions: its members may serve as potential targets to enhance the sensitivity of immunotherapy, while also acting as drivers of immune escape. An in-depth analysis of the precise regulatory nodes of the Let-7 family within the immune microenvironment could provide novel insights for developing a combined “microenvironment remodeling + immunotherapy” strategy. This approach presents a potential direction for intervention, particularly in addressing treatment resistance issues arising from the immunosuppressive microenvironment in GC.

### 4.4 Signal Network Integration and Functional Collaboration of Let-7 Family Target Genes

The regulatory role of the Let-7 family is not isolated; rather, its target genes often form complex signaling networks that collectively influence the biological processes of GC through cross-regulation [76,101]. These target genes are primarily enriched in core pathways such as cell proliferation and metabolism, invasion and metastasis, therapeutic resistance, and immune evasion, all of which collaboratively shape the progression of GC through intersecting regulatory mechanisms.

In the proliferation-metabolism network, the Let-7 family inhibits key hub molecules such as C-Myc and the AKT/mTOR pathway, thereby blocking the cascade of glycolytic metabolic reprogramming and cell growth signals. This establishes a coordinated inhibitory axis of “transcriptional regulation-metabolism-proliferation” [60,94,95,97]. Invasion and metastasis-related targets converge on the epithelial-mesenchymal transition (EMT) regulatory network, which synergistically modulates cytoskeletal remodeling, extracellular matrix degradation, and transcription factor networks to suppress tumor cell migration and invasion [60,77]. Within the therapeutic resistance-immune escape network, Let-7 family members construct an intersecting regulatory framework of “drug response-immune microenvironment-metabolic reprogramming” by regulating genes associated with chemotherapy sensitivity, immune checkpoint molecules, and tumor microenvironmental signals [75,101].

C-Myc and AKT/mTOR serve as critical network hubs that integrate the multifaceted regulatory effects of the Let-7 family. Specifically, C-Myc orchestrates downstream pathways, including glycolytic metabolism and cytoskeletal remodeling [94,97], while AKT/mTOR coordinates growth signaling, autophagic inhibition, and the expression of molecules related to immune escape [60,95]

This highly interconnected network architecture underscores that the tumor-suppressive efficacy of the Let-7 family relies on synergistic multi-target regulation; single-target interventions are often compromised by signaling redundancy. Network-based analyses suggest that combined targeting of core nodes, such as C-Myc/AKT or EMT-associated signaling axes, holds promise for overcoming the limitations of single Let-7 analogs, thereby providing a theoretical foundation for developing multi-target combinatorial therapies in GC.

## 5 Association of the Let-7 Family with Chemotherapy in GC

Chemotherapy is a common treatment modality for various types of cancer; however, most cancers typically exhibit a certain degree of drug resistance [102–104]. Understanding the evolutionary mechanisms of drug resistance in GC is crucial for enhancing the efficacy of chemotherapy and reducing cancer-related mortality. Research indicates that members of the Let-7 family not only predict the response of GC patients to chemotherapy drugs but also directly participate in regulating this response.

### 5.1 Potential of Members of the Let-7 Family as Biomarkers for Predicting Chemotherapy Efficacy in GC

Let-7a has shown potential in evaluating the efficacy of chemotherapy for GC. A study utilizing qRT-PCR demonstrated a significant decrease in Let-7a expression levels in peripheral blood mononuclear cells of patients following neoadjuvant chemotherapy [105]. Additionally, in docetaxel-resistant and sensitive cell lines, Let-7a showed variable expression (both downregulation and upregulation), suggesting its utility as a biomarker for sensitivity to docetaxel treatment [106]. Furthermore, Let-7g-3p expression is associated with the efficacy of neoadjuvant intraperitoneal and systemic chemotherapy, with studies revealing a reduction in its expression post-treatment; low expression levels significantly correlate with negative outcomes in peritoneal metastasis [100]. In hydroxycamptothecin (HCPT)-resistant GC cells, Let-7g and miR-98 display an upregulation trend, and their low expression predicts better efficacy of HCPT chemotherapy, highlighting their predictive value for the effectiveness of the HCPT regimen [107]. Moreover, the overexpression of Let-7f and Let-7g is linked to prolonged time to progression following treatment with cisplatin combined with fluorouracil (CF), suggesting their potential utility in identifying patients who are likely to respond favorably to the CF regimen [108].

Members of the Let-7 family demonstrate considerable potential for application in predicting the efficacy and screening biomarkers for various chemotherapy regimens in GC, as evidenced by their differential expression patterns. The dynamic alterations in Let-7 expression are closely associated with treatment sensitivity, metastasis risk, and disease progression, thereby providing novel molecular targets for the development of individualized chemotherapy regimens.

### 5.2 Regulation of Chemotherapy Sensitization by the Let-7 Family

Let-7b enhances the chemosensitivity of cisplatin/vincristine-resistant GC cell lines (SGC7901/DDP, SGC7901/VCR) by targeting and downregulating C-Myc, indicating its potential as a chemosensitizer [97]. Mechanistically, Let-7b directly targets and regulates Aurora kinase B (AURKB) and X-inactive specific transcript (Xist), thereby inhibiting the growth of cisplatin-resistant cells [109,110]. Furthermore, low expression levels of Let-7i are significantly associated with poor responses to neoadjuvant chemotherapy in GC patients, underscoring its dual role as both a predictive marker for therapeutic efficacy and a regulator of chemosensitivity [69].

These findings indicate that specific members of the Let-7 family regulate the chemosensitivity of GC through distinct molecular mechanisms. When most family members, which act as tumor suppressors, are downregulated, they promote tumor cell proliferation and invasion by activating target genes such as RAB40C and C-Myc, thereby significantly reducing chemosensitivity [55,94]. Conversely, the upregulation of specific members like Let-7g and Let-7d-5p contributes to drug resistance by targeting tumor suppressor genes such as PRDM5 [64]. Furthermore, members of the Let-7 family are intricately involved in the molecular network of chemotherapy resistance, regulating key signaling pathways such as AKT/mTOR and Wnt/β-catenin, which affect the balance of cell apoptosis and autophagy [95]. These mechanisms provide multi-dimensional molecular targets and potential therapeutic strategies for overcoming chemotherapy resistance in GC. The consistent results observed in drug-resistant cell lines and clinical samples further underscore the translational application prospects of these findings.

### 5.3 Mechanisms of Drug Resistance in GC Regulated by Let-7-lncRNA Interaction

Let-7c-3p exhibits a competitive interaction with an lncRNA (NONHSAT160169.1). Elevating the expression of this lncRNA mitigates the inhibitory effect of Let-7c-3p on SRY-box transcription factor 2 (SOX2), leading to increased resistance of GC cells to lapatinib [99]. Additionally, miR-98-5p is negatively correlated with the lncRNA PITPNA-AS1, which is overexpressed in cisplatin-resistant GC cells and tissues. Knockdown of PITPNA-AS1 enhances cisplatin sensitivity in GC cells. Therefore, increasing miR-98-5p levels downregulates PITPNA-AS1, thereby improving cisplatin sensitivity in GC cells [111]. These findings underscore the regulatory role of the “miRNA-lncRNA” axis in drug resistance pathways, offering a novel target for lncRNA-targeted therapies aimed at reversing drug resistance.

Personalized chemotherapy decision-making is of paramount importance in the treatment of GC patients [7]. Research indicates that Let-7 expression not only predicts responses to chemotherapy but also serves as a biomarker for evaluating the sensitivity of GC patients to specific chemotherapy agents. The Let-7 family, as a crucial regulatory factor, facilitates more precise personalized treatment options by modulating mechanisms of chemotherapy sensitization. Moreover, analyzing Let-7 expression profiles enables clinicians to select effective chemotherapy drug combinations more accurately, thereby optimizing treatment regimens. With ongoing research into Let-7’s role in regulating chemotherapy sensitivity in GC, more refined personalized chemotherapy regimens based on Let-7 expression profiles are anticipated in the future.

## 6 Frontier Advances of Let-7 in GC Treatment

### 6.1 Exogenous Interventions for the Regulation of Let-7 Expression and Antigastric Cancer Mechanisms

Modulating the expression levels of the Let-7 family in GC tissues has emerged as a critical approach for optimizing existing therapies and exploring novel treatment strategies. Studies have demonstrated that plant-derived chrysin can alleviate the inhibitory effect on Let-7a, thereby promoting its upregulation in GC tissues [112]. However, the poor water solubility of chrysin limits its bioavailability and clinical application. To overcome this challenge, researchers have employed nanoencapsulation technology. Experimental results indicate that nanoencapsulated chrysin significantly enhances the expression level of Let-7a [113]. A detailed investigation of its mechanism revealed that chrysin induces apoptosis in GC cells by upregulating Let-7a expression and downregulating COPB2 levels, effectively inhibiting cell growth and invasive behavior [96].

Moreover, oleanolic acid, a pentacyclic triterpenoid compound widely present in plants, has emerged as a significant agent in the treatment of GC. Studies have confirmed that oleanolic acid can promote the balance of Treg and Th17 cells by upregulating the expression of miR-98-5p, thereby exerting therapeutic effects on GC [75,114]. In the realm of traditional Chinese medicine, the formula Yang Zheng San Jie Decoction has also demonstrated anticancer potential. Early animal experiments indicated that this formula can upregulate the expression of Let-7a and downregulate the expression of C-Myc in transplanted tumors in nude mice. Subsequently, using the serum pharmacological method of traditional Chinese medicine, serum containing the components of Yang Zheng San Jie Decoction was prepared and applied to human GC cell lines AGS and HS-746T. It was found that the levels of Let-7a in both cell lines significantly increased, while the expression of C-Myc decreased. This further confirms that Yang Zheng San Jie Decoction can inhibit cell proliferation by enhancing the expression of Let-7a in GC cells and induce apoptosis by suppressing its target gene, C-Myc [115].

In studies involving various substances, the functional peptide product soft-shelled turtle peptide, developed based on the nutritional and medicinal value of soft-shelled turtles, demonstrates a regulatory relationship with miRNA expression in GC tissues. Treatment of the human GC cell line AGS with soft-shelled turtle peptide resulted in alterations to the miRNA expression profile, notably increasing the expression of 101 miRNAs, including Let-7d [116]. Additionally, the small-molecule compound anticancer bioactive peptide (ACBP) warrants attention, as it influences Let-7 expression both as a standalone agent and in conjunction with oxaliplatin. The combination of ACBP and oxaliplatin not only exhibits a significant anticancer sensitization effect but also substantially enhances the quality of life in tumor-bearing nude mice while reducing the toxic side effects of chemotherapy drugs on these animals [117]. Table 3 summarizes the therapeutic agents that regulate Let-7 expression and their corresponding molecular mechanisms.

**Table 3:** Therapeutic agents regulating Let-7 expression and their molecular mechanisms

Therapeutic agent	Molecular mechanism	Target
Oleanolic acid [75]	Oleanolic acid upregulates miR-98-5p, which directly targets and suppresses IL-6 expression. This inhibition reverses the Treg/Th17 imbalance by reducing Th17 differentiation and modulating immune suppression in GC.	miR-98-5p, IL-6
Chrysin [96,112,113]	Chrysin downregulates H19, leading to the release of Let-7a, which subsequently inhibits the translation of COPB2, thereby inducing apoptosis in tumor cells and inhibiting their growth. Additionally, it inhibits DNA methyltransferase and histone deacetylase, thereby alleviating the methylation of the Let-7a promoter.	H19, COPB2, DNA methyltransferase, and histone deacetylase
Yang Zheng San Jie Decoction [115]	Upregulates Let-7a expression, which inhibits c-Myc translation, suppressing proliferation and inducing apoptosis.	Let-7a, c-Myc
Soft-shelled turtle peptide [116]	Significantly upregulate the expression of Let-7d-star and restore its tumor suppressive function.	Let-7d
ACBP [117]	ACBP influences Let-7 through regulating Lin28 expression: In SGC7901 and GES-1 cell lines, ACBP-mediated downregulation of Lin28 results in upregulation of Let-7. This process inhibits oncogenes, including c-Myc, and suppresses cell proliferation.	Lin28, Let-7

### 6.2 Research Breakthroughs in Let-7 Targeted Delivery Systems

The delivery of Let-7 into tumors represents a promising therapeutic approach. However, the rapid degradation of miRNA in plasma necessitates further research into delivery methods for Let-7. The selection of appropriate carriers may offer a viable solution. Currently, aptamer-mediated delivery systems have demonstrated significant advantages; for instance, a conjugate of the DNA-targeting aptamer nucleolin-specific aptamer (AptNCL) and Let-7d selectively inhibits the proliferation of GC MKN-45 cells while sparing normal gastric mucosal epithelial cells [118]. Furthermore, a chimera constructed with AptNCL and Let-7d enhances the inhibitory effect on GC cells by suppressing the JAK-2 pathway [119]. In the realm of nanocarrier research, a polyethylene glycol (PEG)-modified nanoparticle system has achieved tumor suppression *in vivo* without significant toxicity through the co-delivery of Let-7 and paclitaxel [120]. This type of carrier not only addresses the stability issues associated with miRNA but also facilitates targeted delivery to tumor cells via surface functionalization modifications, providing an innovative paradigm to overcome the delivery limitations of traditional chemotherapy drugs.

## 7 Conclusion

Accumulating evidence demonstrates that members of the Let-7 family exhibit dysregulated expression in GC and play crucial roles in regulating various biological processes in GC cells, including proliferation, cell cycle progression, migration, stemness, and metabolism. A thorough comprehension of the differential expression patterns of Let-7 in GC, coupled with its comprehensive regulatory network of targets, not only elucidates the specific mechanisms driving GC pathogenesis and progression but also provides a robust theoretical basis and potential targets for innovative therapeutic strategies. Notably, recent studies highlight that Let-7 significantly modulates, and may even dictate, the chemosensitivity of GC, as evidenced by certain drugs exerting anti-GC effects through modulation of Let-7 expression. These findings substantially elevate the therapeutic value of Let-7 as a promising target. However, despite these significant advances, the safe and efficacious translation of Let-7-based miRNA therapy into clinical practice faces substantial hurdles, such as delivery challenges and off-target effects. To realize the safe application of Let-7-based therapies for GC patients, enhance their quality of life, and prolong survival, concerted clinical research efforts are imperative to address these obstacles.

## Data Availability

Not applicable.
